# miR2118 Negatively Regulates Bacterial Blight Resistance through Targeting Several Disease Resistance Genes in Rice

**DOI:** 10.3390/plants12223815

**Published:** 2023-11-09

**Authors:** Xiumei Zhu, Yongjie Kuang, Yutong Chen, Jia Shi, Yaqian Cao, Jixiang Hu, Chao Yu, Fenghuan Yang, Fang Tian, Huamin Chen

**Affiliations:** 1 State Key Laboratory for Biology of Plant Diseases and Insect Pests, Institute of Plant Protection, Chinese Academy of Agricultural Sciences, Beijing 100193, China; ndzhuxiumei@163.com (X.Z.); yongjie_kuang@163.com (Y.K.); cythxmichelle@163.com (Y.C.); qwer1768004860@163.com (J.S.); cyq4537@sina.com (Y.C.); yudoxichao@163.com (C.Y.); fhyang1982@163.com (F.Y.); tfcaas@126.com (F.T.); 2 Jiangsu Coastal Areas Institute of Agricultural Science, Jiangsu Academy of Agricultural Sciences, Yancheng 224002, China; hujixiangvip@163.com

**Keywords:** miR2118, *Xanthomonas oryzae* pv. *oryzae*, bacterial blight, NLR, negative regulation

## Abstract

Plant miRNAs are a class of noncoding RNA with a length of 21–24 nt that play an important role in plant responses to biotic and abiotic stresses. Bacterial blight (BB) caused by *Xanthomonas oryzae* pv. *oryzae* (*Xoo*) is one of the most serious bacterial diseases in rice. Our previous work showed that osa-miR2118b/n was induced by *Xoo* infection. However, the biological function of miR2118 has not yet been characterized in experiments. Herein, we constructed *MIR2118b* OE, as well as single and double mutants of *MIR2118b/n* using CRISPR/Cas9. Further results showed that osa-*MIR2118b* OE plants exhibited longer lesion lengths than the wild type after *Xoo* inoculation, while *MIR2118* CRISPR plants exhibited shorter lesion lengths than the wild type after *Xoo* inoculation. Co-transformation experiments in rice protoplasts indicated that osa-miR2118 negatively regulated the transcripts of three nucleotide-binding sites and leucine-rich repeat (NLR) genes (*LOC_Os08g42700.1*, *LOC_Os01g05600.1*, and *LOC_Os12g37290.1*) which are predicted target genes of miR2118, but not the mutated NLR genes with a 3 bp insertion at the center of the binding sites. The transcriptional level of the three NLR genes was reversed relative to osa-miR2118 in the *MIR2118b* OE and *MIR2118b* CRISPR plants. The above results demonstrate that osa-miR2118b/n negatively regulates the resistance to bacterial blight through negatively regulating several NLR genes.

## 1. Introduction

MicroRNAs (miRNAs) are non-coding RNA molecules 21–24 nucleotides in length [1,2]. They regulate plant growth responses and development [3,4,5] as well as participate in stress resistance and disease resistance [6,7,8] by cleaving complementary target gene mRNA and inhibiting the translation of a target gene or producing small interfering RNA to silence the target gene [9,10,11]. In *Arabidopsis thaliana*, miR156, miR172, miR171, and miR398 regulate the expression of target genes *SPL3* (*squamosa promoter binding-like3, SPL3*), *AP2* (*apetala2*, *AP2*), *SCL4* (*scarecrow-like4*, *SCL4*), and *CSD2* (*Cu/Zn superoxide dismutases2*, *CSD2*), respectively, by inhibiting translation [12,13,14,15,16,17].

miRNAs undergo various changes in expression to regulate the immune response when plants are infected by pathogens [18,19]. Ath-miR393 was the first miRNA reported to play a critical role in plant immune responses. In *Arabidopsis thaliana*, the pathogen-associated molecular pattern (PAMP) flg22 induces the accumulation of miR393. The targets of miR393 are F-box auxin receptors, such as *TIR1*, *AFB2*, and *AFB3*. The inhibition of auxin signaling is associated with enhanced disease resistance in plants because it induces PAMP-triggered immunity (PTI) in plants to resist pathogen infection [20]. Similar to miR393, both miR160 and miR167 can be induced by *Pseudomonas syringae* and flg22 to respond to biological stresses by regulating auxin signals [19,21,22]. However, the expression of other miRNAs, such as miR398a and miR773, decreases during pathogen invasion, thus abolishing the repression of target genes and activating plant PTI [23]. In addition to PTI, many miRNAs directly target the transcription of R genes and participate in effector-triggered immunity (ETI). It has been reported that some miRNAs can guide the cutting of R genes in *Solanaceae*, *Legumes*, and *Arabidopsis*, including miR482/miR2118 and miR6019/miR6020 [24,25,26,27].

The identification of miRNA in plants has been facilitated by RNA cloning, computer algorithm prediction, and high-throughput sequencing. And, the regulatory functions of miRNAs are involved in many biological processes. miRNAs are classified into different families based on their nucleotide sequence, and every family contains one or more members. miR2118 and miR482 have high homology, with partial sequence overlap [28]. Early studies classified them in the same semi-conserved superfamily, and they targeted members of different R gene families in tomatoes, potatoes, soybeans, and medicks to regulate disease resistance in plants [29,30,31]. However, their distribution in plants shows significant specificity, indicating that miR2118 and miR482 play different roles in immune defense in plants and have different effects. miR482 is downregulated during pathogen invasion, and thus abolishes the repression of the target gene and enhances disease resistance in plants [32,33]. Our previous study showed that osa-miR2118b/n of the miR2118 family in rice were induced by *Xanthomonas oryzae* pv. *oryzae* (*Xoo*) infection which causes the most serious bacterial disease, Bacterial Blight of Rice (BB), and no other members of the miR2118 family have changed significantly [34]. However, the biological function of miR2118 in disease resistance has not been identified through genetic experiments.

In this study, we constructed osa-*MIR2118b* overexpression (OE) plants, as well as single and double mutants of *MIR2118b/n*, through CRISPR/Cas9 technology. Furthermore, we carried out disease resistance analysis on *MIR2118b* OE and *MIR2118b/n* CRISPR materials, measured the expression of related genes, and validated the target genes of miR2118. This study verified the regulatory function of miR2118 against *Xoo* infection, and improved our understanding of the regulatory mechanisms of miRNAs in plant immune defense.

## 2. Results

### 2.1. MIR2118 CRISPR Homozygous Line Screening

Previous sRNA high-throughput sequencing showed that the expression of miR2118 was up-regulated in rice after *Xoo* infection, but only osa-miR2118b/n was specifically induced by *Xoo* infection [34]. Therefore, the CRISPR/Cas9 technique was used to specifically knock out the core regions of osa-miR2118b (Chr4: 21645323–21645344) and osa-miR2118n (Chr4: 21661532–21661553) to construct *MIR2118b* ^b^ CRISPR (b locus mutation), *MIR2118n* CRISPR ^c^ (n locus mutation), and *MIR2118b/n* CRISPR ^a^ (double mutations) rice. After the sequencing analysis, we obtained the *MIR2118* CRISPR homozygous mutation lines (Table 1).

### 2.2. MIR2118b OE Material Identification

**Figure 1:**
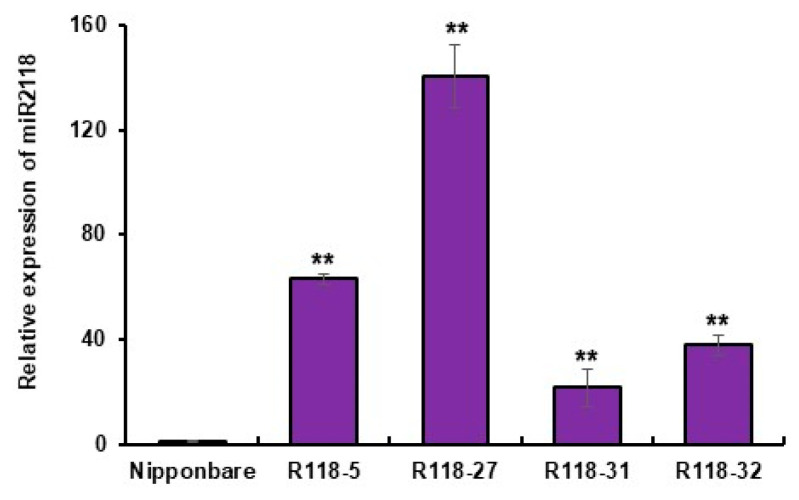
The relative expression of osa-miR2118 in *MIR2118b* OE plants. Nipponbare represents the nontransgenic wild-type plants. R118-5, R118-27, R118-31, and R118-32 represent different lines of *MIR2118b* OE plants. Error bars represent the standard deviations. Asterisks denote a significant difference between transgenic plants and the WT (Student’s *t*-test, ** *p* < 0.01). This experiment was repeated at least three times with similar results.

We constructed a miRNA overexpression binary vector and obtained the *MIR2118b* OE rice under Nipponbare background. The expression level of mature miR2118 was measured using RT-qPCR. The results (Figure 1) showed that the mature miR2118 expression level was much higher in the overexpression plants (R118-5, R118-27, R118-31, and R118-32) than that in wild type, Nipponbare. Thus, R118-5, R118-27, R118-31, and R118-32 are overexpressing transgenic plants with stable osa-miR2118 accumulation.

### 2.3. Disease Resistance Detection

**Figure 2:**
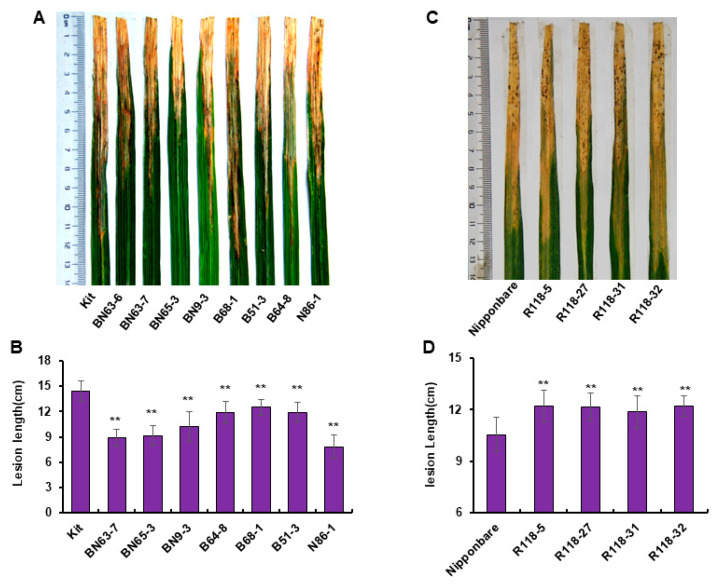
Symptom and lesion length caused by *Xoo* infection in *MIR2118* CRISPR and *MIR2118b* OE plants. Symptoms of inoculated leaves (**A**) and quantitative analysis of lesion lengths (**B**) in *MIR2118* CRISPR plants; symptoms of inoculated leaves (**C**) and quantitative analysis of lesion lengths (**D**) in *MIR2118b* OE plants. *Xoo* strain PXO99^A^ at OD_600_ of 0.5 was inoculated on one-month rice seedlings using the leaf-clipping method. Symptom and lesion length were investigated at 14 days after inoculation. Kit represents the control Kitaake to *MIR2118b/n* CRISPR plants. BN63-6, BN63-7, and BN9-3 represent the double mutant with mutations at both the miR2118b and miR2118n sites; B68-1, B51-3, and B64-8 represent the single mutant with mutation at the miR2118b site; N86-1 represents the single mutant with mutation at the miR2118n site. Nipponbare represents the control of *MIR2118b* OE plants. R118-5, R118-27, R118-31, and R118-32 represent different lines of *MIR2118b* OE plants. Error bars represent the standard deviations. Asterisks denote a significant difference between transgenic plants and the WT (Student’s *t*-test, ** *p* < 0.01). This experiment was repeated at least three times with similar results.

PXO99^A^, a wild-type strain of *Xoo*, was inoculated on the *MIR2118* CRISPR homozygous mutant line and *MIR2118b* OE rice plants. Among the *MIR2118* CRISPR homozygous mutant plants, the lesion lengths of double mutation materials BN63-6, BN63-7, BN65-3, and BN9-3 and n locus mutant N86-1 were much lower than those of the wild-type Kitaake (Kit), and the lesion lengths of b locus mutants B68-1, B51-3, and B64-8 were slightly shorter than those of the wild type (Figure 2A,B), confirming that *MIR2118* CRISPR plants enhance bacterial blight resistance. Mutations at different loci showed that *MIR2118b* and *MIR2118n* genes likely regulate disease resistance in rice. Among *MIR2118b* OE plants, the lesion lengths of R118-5, R118-27, R118-31, and R118-32 were longer than those of wild-type plants, and these differences were significant (*p* < 0.01). Thus, the overexpression of *MIR2118b* increased the susceptibility to bacterial blight (Figure 2C,D).

### 2.4. Validation of the Target Genes of miR2118

#### 2.4.1. Construction of Related Vectors

**Figure 3:**
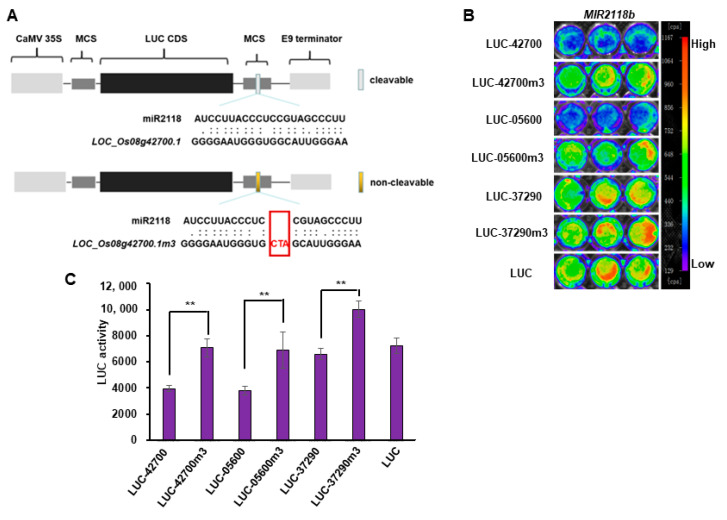
Validation of the inhibition of miR2118b to candidate targets via transient expression in rice protoplasts. (**A**) Diagram of the construction of LUC-42700 and LUC-42700m3; The red CTA in box was inserted into the binding sites between 10-11 bases to interrupt the specific binding between miRNA and target mRNA. (**B**) fluorescence intensity after the co-transformation of pUC-*MIR2118* with different LUC fusion vectors in the rice protoplast system; (**C**) quantitative analysis of LUC activity in (**B**). Error bars represent the standard deviations. Asterisks denote a significant difference between co-transformation with LUC-fusion and with LUC-fusion mutation (Student’s *t*-test, ** *p* < 0.01). This experiment was repeated at least three times with similar results.

psRNATarget software [35] was used to predict the target genes of miR2118, and three target genes (*LOC_Os08g42700.1*, *LOC_Os01g05600.1*, and *LOC_Os12g37290.1*) related to resistance were selected for further validation. To validate the above candidate targets of miR2118 in protoplast transient expression, related target fusion vectors and target mutation fusion vectors were constructed according to the presentation in Figure 3A. As miR2118 was binding to the 3′UTR of *LOC_Os08g42700.1*, we inserted 22 bases of *LOC_Os08g42700.1* that were complementary to miR2118 into the 3′UTR of the LUC gene to obtain the target gene reporter vector pUC-LUC-42700. miRNAs bind to target genes via base complementation. Moreover, the core region of miRNAs is at the 9-11 bases. Therefore, three bases of CTA were inserted into the 10–11 bases of the target loci. This maximally disrupted the sequence complementation between miR2118 and its target gene *LOC_Os08g42700.1*, causing miR2118 to be unable to pair with its target gene and thus unable to carry out cleavage. This modified sequence was inserted into the 3′UTR of the LUC gene to obtain the target gene mutant reporter vector, pUC-LUC-42700m3. pUC-LUC-05600, pUC-LUC-05600m3, pUC-LUC-37290, and pUC-LUC-37290m3 were obtained using the same method.

#### 2.4.2. Protoplast Transient Expression for Target Gene Validation

Plasmid pUC-*MIR2118* was mixed separately with plasmid pUC-LUC-42700, pUC-LUC-05600, pUC-LUC-37290, pUC-LUC-42700m3, pUC-LUC-05600m3, pUC-LUC-37290m3, and control pUC-LUC and co-transfected into protoplasts. At 18 h after incubation, the plant live imaging system was used to measure fluorescence intensity, and a luminometer was used to measure LUC activity. The fluorescence intensity in the pUC-*MIR2118* and pUC-LUC-42700 co-transfected reaction was weaker than that in pUC-*MIR2118* and pUC-LUC reaction, or the pUC-*MIR2118* and pUC-LUC-42700m3 reaction (Figure 3B). The quantitative analysis of LUC relative activity was also consistent with the fluorescence intensity results (Figure 3C). *MIR2118* expression inhibits the LUC activity of pUC-LUC-42700 but does not inhibit the LUC activity of pUC-LUC-42700m3. This result showed that miR2118b can repress the transcription of *LOC_Os08g42700.1* through sequence complementation specificity. Similarly, *MIR2118* expression significantly inhibits the LUC activity of pUC-LUC-05600 but not pUC-LUC-05600m3, and inhibits the LUC activity of pUC-LUC-37290 but not pUC-LUC-37290m3 (Figure 3B,C). These results suggested that miR2118 specifically represses *LOC_Os08g42700.1*, *LOC_Os01g05600.1*, and *LOC_Os12g37290.1*.

#### 2.4.3. Target Gene Expression Level Analysis of Transgenic Materials

**Figure 4:**
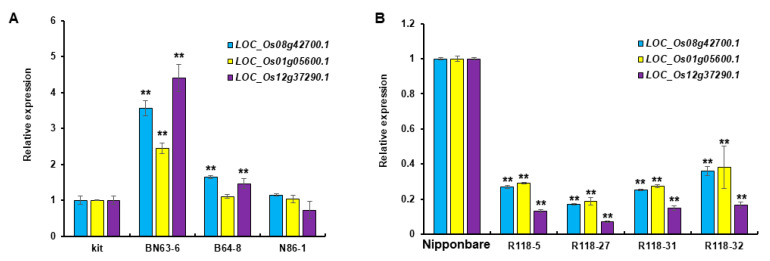
The transcriptional levels of target genes in *MIR2118* CRISPR (**A**) and *MIR2118b* OE (**B**) plants. Kit represents the control Kitaake to *MIR2118b/n* CRISPR plants. BN63-6 represents the double mutant with mutations at both the miR2118b and miR2118n sites; B64-8 and N86-1 represent the single mutant with mutation at the miR2118b and miR2118n site, respectively. Nipponbare represents the control to *MIR2118b* OE plants. R118-5, R118-27, R118-31, and R118-32 represent the different lines of *MIR2118b* OE plants. Error bars represent the standard deviations. Asterisks denote a significant difference between transgenic plants and the WT (Student’s *t*-test, ** *p* < 0.01). This experiment was repeated at least three times with similar results.

To further validate the target genes of miR2118, the transcriptional level of *LOC_Os08g42700.1*, *LOC_Os01g05600.1*, and *LOC_Os12g37290.1* were measured in *MIR2118* CRISPR homozygous lines and *MIR2118b* OE plants. The results showed that the transcripts of target genes were higher in b locus mutant than those in the wild type, Kitaake, and much higher in the bn loci mutant. It is a pity that the transcripts of three NLR genes did not show a significant difference between N86-1 (n locus mutant) and the wild type *(*Figure 4A). Based on the transcripts of target genes in *MIR2118b/n* CRISPR (double mutants) and *MIR2118b* CRISPR (single mutant), we speculate that miR2118b and miR2118n function in a synergistic way. Thus, mutant miR2118b/n abolishes target gene repression. Consistently, the transcripts of target genes *LOC_Os08g42700.1*, *LOC_O s01g05600.1*, and *LOC_Os12g37290.1* were much lower in *MIR2118b* OE lines than those in the wild type, Nipponbare *(*Figure 4B). Thus, the overexpression of miR2118 decreases the expression of resistance-associated genes. The above results showed that three NLR genes were the target genes of miR2118.

## 3. Discussion

Many miRNAs have been discovered with the application of high-throughput sequencing techniques in maize [36,37], *Arabidopsis* [8], soybean [38], rice [39], and other plants. miRNAs are classified into different families based on their nucleotide sequence, with each family containing one or more members. However, the discovery of new miRNAs in plants has stabilized, and the new challenges include identifying miRNAs with species- or tissue-specific expression, discovering the biological function of miRNA, and validating the real targets of miRNA as well.

The rice miR2118 family contains 18 precursor genes that generate 12 mature miRNAs with a length of 22 nt (miRBase: http://www.mirbase.org/, accessed on 15 August 2018). In 2009, Jagadeeswaran identified four new Fabaceae-specific miRNAs by aligning sequencing results with the *Medicago truncatula* genome and expressed sequences tag (EST) resources, among which one was miR2118. The rapid amplification of cDNA ends (RACE) was used to validate that three *TIR-NBS-LRR* coding genes (*AC202360_18.1*, *AC203224_17.1*, and *AC143338_38.2*) are targets of the newly identified miR2118 [25]. Subsequently, the existence of miR2118 was also confirmed in monocotyledonous maize and rice [40]. Furthermore, miR2118 can respond to a variety of environmental stresses. For instance, miR2118 was up-regulated by abiotic stress, particularly drought and abscisic acid stress in the legume *phaseolus vulgaris* [41]. miR2118 regulates drought resistance through repressing disease resistance genes *CiDR1* and *CiDR2* in *Caragana intermedia* [42]. In addition, miR2118 responds to cold stress in wheat [43].

Even though many plant miRNAs are regulated by pathogens, we understand less about the functions of miRNA in plant disease resistance than their role in plant development. miR482 and miR2118 belong to the same family and have similar sequences, and the target genes are mainly NLR genes [28,29]. miR482 are downregulated after virus and fungal infection in tomato and cotton, which abolishes their inhibitory effects on NLRs and demonstrates the active defensive behavior of the host towards pathogen infection [26,33]. In our previous works, we found osa-miR2118b/n, but not other isoforms of the miR2118 family, to be upregulated by *Xoo* infection in rice [34]. However, the biological function of miR2118 has not yet been identified through experiments so far.

In this study, we constructed *MIR2118b* OE and *MIR2118b/n* CRISPR rice, respectively. Further disease resistance results showed that *MIR2118b* OE plants were more susceptible to BB, while *MIR2118b/n* CRISPR plants were more resistant to BB. In addition, three miR2118 target NLR genes were validated according to the expression levels of potential target genes in *MIR2118b/n* CRISPR and *MIR2118b* OE plants, as well as the specific effects of miR2118 and predicted target gene-binding sites. Because of the stronger resistance and lower transcriptional levels of NLR genes in *MIR2118b/n* CRISPR (double mutants) than in the wild type and *MIR2118b* or *MIR2118n* CRISPR (single mutant), we predicted that miR2118b and miR2118n would function in a synergistic way. Although miR2118 regulates the resistance to BB in a modest way, our results solidly indicated that miR2118 negatively regulates the resistance to BB. Therefore, we concluded that miR2118 negatively regulates bacterial blight resistance through targeting several disease resistance genes in rice. Combined with the induced expression of miR2118b/n by *Xoo* infection, we hypothesized that the pathogenic bacteria may recruit miR2118b/n to enhance the repression of NLR genes and facilitate bacterial infection. However, little is known about what the regulatory mechanism of bacterial infection on miR2118 expression is. Further study is still required to fully understand the molecular mechanism of miR2118 in rice–*Xoo* interactions.

## 4. Materials and Methods

### 4.1. Rice Materials

For *MIR2118b* OE material [44], *MIR2118b* fragment (312bp length) was amplified from rice genomic DNA using specific primer *MIR2118b*-F and *MIR2118b*-R, and inserted into pCXUN (digested with *Xcm* I) [45] to obtain pCXUN-*MIR2118b*. And, pCXUN-*MIR2118b* was transformed into Nipponbare through *Agrobacterium*-mediated transformation and obtained *MIR2118b* OE rice.

For *MIR2118* CRISPR materials [46], the 19–20 bp complementary oligos initialized by “G”, and gRNAB-1 AGGAGCAC-CTAAGAACGAT, gRNAB-2 AGGAGCACCTAAGAACGAT were designed for mutation miR2118b sites, and gRNAn-1 ATGCCTCCCATTCCTATCG and gRNAn-2 CTTCCTTTCAGCTCGCACG were designed for mutation miR2118n sites, respectively. Moreover, all oligos should carry the appropriate 4-bp adaptor, and were phosphorylated and annealed. gRNAB-1 was inserted into the *BtgZ* I-digested pENTR4: sgRNA4; gRNAB-2 was inserted into the *Bsa* I-digested pENTR4: sgRNA4; gRNAn-1 was inserted into the *BtgZ* I-digested pENTR4: sgRNA5; gRNAn-2 was inserted into the *Bsa* I-digested pENTR4: sgRNA5. Each gRNA expression cassette was shuffled into the binary vector pUbi: SpCas9 using LR clonase (Invitrogen, Carlsbad, CA, USA). gRNAB and gRNAn were inserted into Kitaake singly or combinedly through *Agrobacterium*-mediated transformation, and detailed mutation information was analyzed after sequence genotyping.

Rice seeds were disinfected with 75% alcohol and spread in 1/2MS medium. (1) Rice materials prepared for the protoplasmic system were cultured in an incubator for 10 days. (2) Transgenic plants were used for phenotypic detection. After the rice germinated to about 5 cm, the rice seedlings were transferred to a rice culture medium, were grown to 20 cm, and were planted in a glass greenhouse for about six weeks for phenotypic detection. 

### 4.2. RNA Extraction, Reverse Transcription, and RT-qPCR Analysis

The total RNA of rice leaves was extracted using the TRIzol method, and the genomic DNA was eliminated using DNase I and then reverse-transcribed into cDNA with a reverse transcription kit (Beijing TransGen Biotech Company, Beijing, China). An ABI-7500 fluorescence quantitative PCR instrument (Thermo Fisher Scientific, Waltham, MA, USA) was used for quantitative detection. The RT-qPCR system was as follows: 2×RealStar Green Fast Mixture with ROX 10 µL, diluted cDNA 5 µL, positive and negative primers 1 µL each, and finally supplemented with RNase-free ddH2O to 20 µL. The procedure of RT-qPCR was as follows: pre-denaturation at 95 °C for 2 min, denaturation at 95 °C for 15 s, annealing at 60 °C for 30 s, amplification for 40 cycles. The gene expression was calculated according to the relative threshold method (2^−∆∆ct^). Three replicates were performed for each sample, and the average value of the three replicates was selected. Multiple comparisons and one-way analysis of variance were performed using GraphPad Prism software (2018).

### 4.3. Disease Resistance Detection

The PXO99^A^ strain was cultured on the PSA plate, and the single colony was selected and cultured in M210 liquid medium, and then transferred to M210 liquid medium for overnight shook at the ratio of 1:100, and cultured to OD_600_ of 0.8–1.0. The bacteria were collected and re-suspended with ddH_2_O to OD_600_ of 0.5. PXO99^A^ was inoculated on rice leaves via the leaf clipping method, and the lesion lengths were measured at 14 days after inoculation [34].

### 4.4. Prediction of Target Genes

psRNATarget software [35] was used to predict the target genes of miR2118, and three target genes related to resistance, namely *LOC_Os08g42700.1*, *LOC_Os01g05600.1* and *LOC_Os12g37290.1*, were listed (Table 2).

### 4.5. Construction of Vector

Using the genome of Nipponbare as the template, the *MIR2118* fragment was amplified with primer *MIR2118*-F and *MIR2118*-R (Table 3) respective containing *BamH* I and *Pst* I restriction site using PCR instrument (Bio-Rad T100™, Bio-Rad Laboratories, Inc., Foster, CA, USA). The pUC19-LUC1 plasmid and the *MIR2118b* fragment were digested with *BamH* I and *Pst* I, ligated, and transformed into *E. coli*. Positive clones were screened and confirmed by sequencing as pUC-*MIR2118b* vector.

A total of 10 µL primer F and primer R were mixed in a 1.5 mL centrifuge tube and incubated in boiled water for 5 min, and cooled in room temperature slowly. The primer mixture (double chains) was ligated with the digested pUC-LUC plasmid. The target gene-LUC fusion expression vector could then be obtained. Three bases were inserted in the middle of the tenth base of miR2118 binding to the target gene to form the target gene mutant.

### 4.6. Rice Protoplast Transient Expression System

The rice protoplast transient expression system for the miRNA verification of target genes established in our laboratory was used to verify whether the target genes were really regulated by miR2118. According to the reports of Chen and He [47,48], rice protoplasts were prepared via enzymolysis, and PEG4000 mediated the transformation of rice cells via related plasmids. For the specific experimental steps of rice protoplast preparation and transformation, refer to Hu [49].

## Figures and Tables

**Table 1:** The information of *MIR2118* CRISPR homozygous mutation lines.

	b Locus	n Locus
BN63-6 ^a^	Del 9 nt (21645316-5324)/Ins 1 nt (21645336-)/ del 4 nt (21645381-5384)	Del 45 nt (21661545-1589)
BN9-3 ^a^	Del 5 nt (21645316-5320)/Ins 1 nt (21645336-)/ Ins 1 nt (21645381-)	Del 2 nt (21661543-1544)
BN65-3 ^a^	Del 29 nt (21645294-5322)/Ins 1 nt(21645336-)/ Ins 1 nt (21645382-)	Ins 1 nt (21661545-)
B51-3 ^b^	Ins 210 nt (21645321-)/del 1 nt (21645321-)/ del 38 nt (21645348-5385)	
B64-8 ^b^	Del 23 nt (21645306-5328)/Ins 1 nt (21645381-)	
B68-1 ^b^	Ins 1 nt (21645320-)/Ins 1 nt (21645382-)	
N86-1 ^c^		Del 27 nt (21661533-1559)/Ins 7 nt (21661532-)

^a^ *MIR2118b/n* CRISPR (double mutations); ^b^ *MIR2118b* CRISPR (b locus mutation); ^c^ *MIR2118n* CRISPR (n locus mutation).

**Table 2:** The predicted target genes of osa-miR2118b/n (psRNAtarget 2017).

Target Gene	Sequence	Inhibition	Target Description
*LOC_Os08g42700.1*	GGGGAAUGGGUG GCAUUGGGAA	Cleavage	cDNA|resistance protein, putative, expressed
*LOC_Os01g05600.1*	AGGGGAUGGGAG GCAUCGGUAA	Cleavage	cDNA|NBS-LRR disease resistance protein, putative, expressed
*LOC_Os12g37290.1*	GGGGAAUGGGU GGUAUUGGGAA	Cleavage	cDNA|resistance protein T10rga2-1A, putative, expressed

**Table 3:** Primer sequences in this work.

Primer	Sequence (5′-3′)	Restriction Enzyme	Purpose
*MIR2118*-F	GGATCCTATACGC ATGGTGTTTCACTTAC	*BamH* I, *Pst* I	Amplified *MIR2118* gene
*MIR2118*-R	CTGCAGTTTAGAGGG ACAGAAGAGTTTGA		
42700-F	tcgacGGGGAATGGGTG GCATTGGGAActgca	*Pst* I, *Sal* I	Construction of LUC-42700 fusion vector
42700-R	gTTCCCAATGCCACCC ATTCCCCg		
42700m3-F	tcgacGGGGAATGGGTG ctaGCATTGGGAActgca	*Pst* I, *Sal* I	Construction of LUC-42700m3 fusion vector
42700m3-R	gTTCCCAATGCtagCACCCATTCCCCg		
05600-F	tcgacAGGGGATGGGAGGCATCGGTAActgca	*Pst* I, *Sal* I	Construction of LUC-05600 fusion vector
05600-R	gTTACCGATGCCTCCCA TCCCCTg		
05600m3-F	tcgacAGGGGATGGGAG ctaGCATCGGTAActgca	*Pst* I, *Sal* I	Construction of LUC-05600m3 fusion vector
05600m3-R	gTTACCGATGC tagCTCCCATCCCCTg		
37290-F	tcgacGGGGAAUGGGU GGUAUUGGGAActgca	*Pst* I, *Sal* I	Construction of LUC-37290 fusion vector
37290-R	gTTCCCAATACCACC CATTCCCCg		
37290m3-F	tcgacGGGGAAUGGGUG ctaGUAUUGGGAActgca	*Pst* I, *Sal* I	Construction of LUC-37290m3 fusion vector
37290m3-R	gTTCCCAATA tagCCACCCATTCCCCg		

## Data Availability

Data are contained within the article.

## References

[1] Ambros V. (2004). The functions of animal microRNAs. Nature.

[2] Reinhart B.J., Weinstein E.G., Rhoades M.W., Bartel B., David P.B. (2002). MicroRNAs in plants. Gene Dev..

[3] Borges F., Martienssen R.A. (2015). The expanding world of small RNAs in plants. Nat. Rev. Cell. Biol..

[4] Cuperus J.T., Fahlgren N., Carrington J.C. (2011). Evolution and Functional Diversification of miRNA Genes. Plant Cell.

[5] Moran Y., Agron M., Praher D., Technau U. (2017). The evolutionary origin of plant and animal microRNAs. Nat. Ecol. Evol..

[6] Khraiwesh B., Zhu J.K., Zhu J.H. (2012). Role of miRNAs and siRNAs in biotic and abiotic stress responses of plants. Biochim. Biophys. Acta.

[7] Shukla L.I., Chinnusamy V., Sunkar R. (2008). The role of microRNAs and other endogenous small RNAs in plant stress responses. Biochim. Biophys. Acta.

[8] Liang G., He H., Yu D.Q. (2012). Identification of Nitrogen Starvation-Responsive MicroRNAs in *Arabidopsis thaliana*. PLoS ONE.

[9] Bartel D.P. (2004). MicroRNAs: Genomics, Biogenesis, Mechanism, and Function. Cell.

[10] Xie Z.X., Allen E., Wilken A., Carrington J.C. (2005). DICER-LIKE 4 functions in trans-acting small interfering RNA biogenesis and vegetative phase change in *Arabidopsis thaliana*. Proc. Natl. Acad. Sci. USA.

[11] Addo-Quaye C., Eshoo T.W., Bartel D.P., Axtell M.J. (2008). Endogenous siRNA and miRNA targets identified by sequencing of the *Arabidopsis* degradome. Curr. Biol..

[12] Aukerman M.J., Sakai H. (2003). Regulation of flowering time and floral organ identity by a microRNA and its *APETALA2*-like target genes. Plant Cell.

[13] Chen X.M. (2004). A microRNA as a translational repressor of *APETALA2* in *Arabidopsis* flower development. Science.

[14] Gandikota M., Birkenbihl R.P., Hohmann S., Cardon G.H., Saedler H., Huijser P. (2007). The miRNA156/157 recognition element in the 3′ UTR of the *Arabidopsis* SBP box gene SPL3 prevents early flowering by translational inhibition in seedlings. Plant J..

[15] Li S.B., Liu L., Zhuang X.H., Yu Y., Liu X.G., Cui X., Ji L.J., Pan Z.Q., Cao X.F., Mo B.X. (2013). MicroRNAs inhibit the translation of target mRNAs on the endoplasmic reticulum in *Arabidopsis*. Cell.

[16] Schwab R., Palatnik J.F., Riester M., Schommer C., Schmid M., Weigel D. (2005). Specific effects of microRNAs on the plant transcriptome. Dev. Cell.

[17] Brodersen P., Sakvarelidze-Achard L., Bruun-Rasmussen M., Dunoyer P., Yamamoto Y.Y., Sieburth L., Voinnet O. (2008). Widespread translational inhibition by plant miRNAs and siRNAs. Science.

[18] Baldrich P., Segundo B.S. (2016). MicroRNAs in Rice Innate Immunity. Rice.

[19] Li Y., Zhang Q.Q., Zhang J., Wu L., Qi Y., Zhou J.M. (2010). Identification of microRNAs involved in pathogen-associated molecular pattern-triggered plant innate immunity. Plant Physiol..

[20] Navarro L., Dunoyer P., Jay F., Arnold B., Dharmasiri N., Estelle M., Voinnet O., Jones J.D.G. (2006). A plant miRNA contributes to antibacterial resistance by repressing auxin signaling. Science.

[21] Franco-Zorrilla J.M., Valli A., Todesco M., Todesco M., Mateos I., Puga M.I., Rubio-Somoza I., Leyva A., Weigel D., García J.A. (2007). Target mimicry provides a new mechanism for regulation of microRNA activity. Nat. Genet..

[22] Fahlgren N., Howell M.D., Kasschau K.D., Chapman E.J., Sullivan C.M., Cumbie J.S., Givan S.A., Law T.F., Grant S.R., Dangl J.L. (2007). High throughput sequencing of Arabidopsis microRNAs: Evidence for frequent birth and death of *MIRNA* genes. PLoS ONE.

[23] Campo S., Cristina P.P., Christelle S., Moreno A.B., Donaire L., Zytnicki M., Notredame C., César L., Segundo B.S. (2013). Identification of a novel microRNA (miRNA) from rice that targets an alternatively spliced transcript of the Nramp6 (Natural resistance-associated macrophage protein 6) gene involved in pathogen resistance. New Phytol..

[24] Padmanabhan C., Zhang X., Jin H. (2009). Host small RNAs are big contributors to plant innate immunity. Curr. Opin. Plant Biol..

[25] Jagadeeswaran G., Zheng Y., Li Y.F., Lata I., Fangshukla L.I., Matts J., Hoyt P., Macmil S.L., Wiley G.B., Roe B.A. (2009). Cloning and characterization of small RNAs from *Medicago truncatula* reveals four novel legume-specific microRNA families. New Phytol..

[26] Shivaprasad P.V., Chen H.M., Patel K., Bond D.M., Santos B.A.C.M., Baulcombe D.C. (2012). A MicroRNA superfamily regulates nucleotide binding site-leucine-rich repeats and other mRNAs. Plant Cell.

[27] Boccara M., Sarazin A., Thiébeauld O., Jay F., Voinnet O. (2014). The *Arabidopsis* miR472-RDR6 silencing pathway modulates PAMP- and effector-triggered immunity through the post-transcriptional control of disease resistance genes. PLoS Pathog..

[28] Zhai J.X., Jeong D.H., De P.E., Park S., Rosen B.D., Li Y., Gonzalez A.J., Yan Z., Kitto S.L., Grusak M.A. (2011). MicroRNAs as master regulators of the plant *NB-LRR* defense gene family via the production of phased, trans-acting siRNAs. Gene Dev..

[29] Li F., Pignatta D., Bendix C., Brunkard J.O., Cohn M.M., Tung J., Sun H.Y., Kumar P., Baker B. (2012). MicroRNA regulation of plant innate immune receptors. Proc. Natl. Acad. Sci. USA.

[30] Vries S.D., Kloesges T., Rose L.E. (2015). Evolutionarily Dynamic, but Robust, Targeting of Resistance Genes by the miR482/2118 Gene Family in the *Solanaceae*. Genome Bio. Evol..

[31] Yin Z.J., Li Y., Han X.L., Shen F.F. (2012). Genome-wide profiling of miRNAs and other small non-coding RNAs in the verticillium dahliae-inoculated cotton roots. PLoS ONE.

[32] Vries S.D., Kukuk A., Von Dahlen J.K., Schnake A., Kloesges T., Rose L.E. (2018). Expression profiling across wild and cultivated tomatoes supports the relevance of early miR482/2118 suppression for *Phytophthora* resistance. Proc. R. Soc. B.

[33] Zhu Q.H., Fan L., Liu Y., Xu H., Llewellyn D., Wilson I. (2013). miR482 regulation of *NBS-LRR* defense genes during fungal pathogen infection in Cotton. PLoS ONE.

[34] Yu C., Chen Y.T., Cao Y.Q., Chen H.M., Wang J.C., Bi Y.-M., Tian F., Yang F.H., Rothstein S.J., Zhou X.P. (2018). Overexpression of miR169o, an overlapping microRNA in response to both nitrogen limitation and bacterial infection, promotes nitrogen use efficiency and susceptibility to bacterial blight in rice. Plant Cell Physiol..

[35] Dai X., Zhuang Z., Zhao P.X. (2018). psRNATarget: A plant small RNA target analysis server (2017 release). Nucleic Acids Res..

[36] Thiebaut F., Rojas C.A., Grativol C., Motta M.R., Vieira T., Regulski M., Martienssen R.A., Farinelli L., Hemerly A.S., Ferreira-Paulo C.G. (2014). Genome-wide identification of microRNA and siRNA-responsive to endophytic beneficial diazotrophic bacteria in maize. BMC Genom..

[37] Zhao M., Tai H., Sun S., Zhang F., Xu Y., Li W.X., Sebastien P. (2012). Cloning and characterization of maize miRNAs involved in responses to nitrogen deficiency. PLoS ONE.

[38] Xu F., Liu Q., Chen L.Y., Kuang J.B., Walk T., Wang J.X., Liao H. (2013). Genome-wide identification of soybean microRNAs and their targets reveals their organ-specificity and responses to phosphate starvation. BMC Genom..

[39] Guo W.X., Wu G.T., Yan F., Lu Y.W., Zheng H.Y., Lin L., Chen H.R. (2012). Identification of novel *Oryza sativa* miRNAs in deep sequencing-based small RNA libraries of rice infected with *Rice stripe virus*. PLoS ONE.

[40] Johnson C., Kasprzewska A., Tennessen K., Fernandes J., Nan G.L., Walbot V., Sundaresan V., Vance V., Bowman L.H. (2009). Clusters and superclusters of phased small RNAs in the developing inflorescence of rice. Genome Res..

[41] Arenas-Huertero C., Perez B., Rabanal F., Blanco-Melo D., Rosa C.D.L., Estrada-Navarrete G., Sanchez F., Covarrubias A.A., Reyes J.L. (2009). Conserved and novel mirnas in the legume *Phaseolus vulgaris* in response to stress. Plant Mol. Biol..

[42] Wu B.F., Li W.F., Han S.Y., Xu H.Y., Qi L.W. (2015). Role of cin-miR2118 in drought stress responses in Caragana intermedia and Tobacco. Gene.

[43] Zhang R.Z., Huang S., Li S.M., Song G.Q., Li G.Y. (2020). Evolution of phas loci in the young spike of allohexaploid wheat. BMC Genom..

[44] Chen Y.Y. (2015). Characterize the function of candidate overlapping miRNAs response to *Xanthomonas oryzae* pv. *oryzae* infection and nitrogen starvation stress in Rice. Chinese Academy of Agricultural Sciences Dissertation. April, 2015 (In Chinese). CAAS. https://d.wanfangdata.com.cn/thesis/ChJUaGVzaXNOZXdTMjAyMzA5MDESCFkyNzg4MDIxGghmY29qcWxzNA%3D%3D.

[45] Chen S., Songkumarn P., Liu J., Wang G.L. (2009). A versatile zero background T-vector system for gene cloning and functional genomics. Plant Physiol..

[46] Zhou H.B., Liu B., Weeks D.P., Spalding M.H., Yang B. (2014). Large chromosomal deletions and heritable small genetic changes induced by CRISPR/Cas9 in rice. Nucleic Acids Res..

[47] Chen S., Tao L.Z., Zeng L.R., Vega-Sanchez M.E., Umemura K., Wang G.L. (2006). A highly efficient transient protoplast system for analyzing defence gene expression and protein–protein interactions in rice. Mol. Plant Pathol..

[48] He F., Chen S.B., Ning Y.S., Wang G.L. (2016). Rice (*Oryza sativa*) protoplast isolation and its application for transient expression analysis: Rice protoplast isolation and transient expression analysis. Curr. Protoc. Plant Biol..

[49] Hu J.X., Cao Y.Q., Zhu X.M., Yu C., Tian F., Yang F.H., Chen H.M., He C.Y. (2019). Rapid validation of target rice miRNAs genes in transient expression system. Biotechnol. Bull..

